# Obinutuzumab-atezolizumab-lenalidomide for the treatment of patients with relapsed/refractory follicular lymphoma: final analysis of a Phase Ib/II trial

**DOI:** 10.1038/s41408-021-00539-8

**Published:** 2021-08-20

**Authors:** Franck Morschhauser, Nilanjan Ghosh, Izidore S. Lossos, M. Lia Palomba, Amitkumar Mehta, Olivier Casasnovas, Don Stevens, Sudhakar Katakam, Andrea Knapp, Tina Nielsen, Ron McCord, Gilles Salles

**Affiliations:** 1 University of Lille, CHU Lille, ULR 7365 - GRITA - Groupe de Recherche sur les formes Injectables et les Technologies Associées, Lille, France; 2 Hematologic Oncology and Blood Disorders, Levine Cancer Institute/Atrium Health, Charlotte, NC USA; 3 Division of Hematology, Department of Medicine, Sylvester Comprehensive Cancer Center, University of Miami, Miami, FL USA; 4 Department of Medicine, Memorial Sloan Kettering Cancer Center, New York, NY USA; 5 Division of Hematology and Oncology, University of Alabama School of Medicine, Birmingham, AL USA; 6 Service d’Hématologie Clinique, CHU Dijon Bourgogne – Hôpital François Mitterrand, Dijon, France; 7 Norton Cancer Institute, Norton Healthcare, Louisville, KY USA; 8 Product Development Oncology, F. Hoffmann-La Roche Ltd, Basel, Switzerland; 9 Genentech Inc., South San Francisco, CA USA; 10 Haematology Department, Université Claude Bernard de Lyon, Lyon University Hospital, Pierre Benite, France

**Keywords:** Immunotherapy, Public health

## Abstract

We evaluated the triplet regimen obinutuzumab-atezolizumab-lenalidomide (G-atezo-len) for patients with relapsed/refractory (R/R) follicular lymphoma (FL) in an open-label, multicenter phase Ib/II study (BO29562; NCT02631577). An initial 3 + 3 dose‐escalation phase to define the recommended phase II dose of lenalidomide was followed by an expansion phase with G-atezo-len induction and maintenance. At final analysis, 38 patients (lenalidomide 15 mg, *n* = 4; 20 mg, *n* = 34) had completed the trial. Complete response rate for the efficacy population (lenalidomide 20 mg, *n* = 32) at end-of-induction was 71.9% (66.7% in double‐refractory patients [refractory to rituximab and alkylator] [*n* = 12]; 50.0% in patients with progressive disease within 24 months of first-line therapy [*n* = 12]). The 36-month progression-free survival rate was 68.4%. All treated patients had ≥1 adverse event (AE; grade 3–5 AE, 32 patients [84%]; serious AE, 18 patients [47%]; AEs leading to discontinuation of any study drug, 11 patients [29%]). There were 2 fatal AEs (1 merkel carcinoma, 1 sarcomatoid carcinoma; both unrelated to any study drug). The G‐atezo-len regimen is effective and tolerable in patients with R/R FL. AEs were consistent with the known safety profile of the individual drugs.

## Introduction

Follicular lymphoma (FL) is the most common indolent non-Hodgkin’s lymphoma (NHL) in the Western world [1]. Although the vast majority of patients treated for FL usually respond to initial chemoimmunotherapy regimens [2], most will ultimately relapse, and experience increasing refractoriness to subsequent lines of therapy [3]. This has led to research into novel treatment regimens such as phosphoinositide 3-kinase inhibitors (PI3K); [4, 5] or those combining an anti-CD20 monoclonal antibody (mAb) and an immunomodulatory agent.

Lenalidomide is an orally active immunomodulatory agent with direct anti-tumor activity as well as indirect effects mediated through T-cell and natural killer (NK) cell function [6]. Specifically, lenalidomide promotes degradation of the hematopoietic transcription factors Ikaros and Aiolos, leading to apoptosis of neoplastic B cells [7–9]. Adding lenalidomide to rituximab has been reported to enhance anti-tumor activity by reversing or reducing the impairment in tumor‐infiltrating T-cell immunologic synapse formation present in patients with FL [10, 11]. In phase II/III studies in patients with NHL, including those with relapsed/refractory (R/R) FL, lenalidomide in combination with rituximab (R2 regimen) demonstrated manageable safety and superior efficacy over rituximab alone [12–16]. Furthermore, chemotherapy-free induction and maintenance treatment with the novel glycoengineered humanized type II anti-CD20 antibody obinutuzumab plus lenalidomide also showed favorable activity and tolerable safety in patients with R/R FL in the phase II GALEN study [17, 18].

Since FL has been considered as particularly immune responsive, further targeting of the immune microenvironment may be beneficial [19, 20]. In patients with FL, programmed death-ligand 1 (PD-L1) is expressed on tumor-infiltrating lymphocytes, macrophages, peripheral blood T cells, and monocytes, but not on tumor cells [21]. Although the impact of lenalidomide on programmed death 1 (PD-1)/PD-L1 expression has not been specifically reported in patients with FL, it has been reported to downregulate PD-L1 expression on plasma cells and to downregulate PD-1 expression on T cells in multiple myeloma [22]. Furthermore, it has been observed that activated NK cells express PD-1 and that PD-L1 engagement could suppress NK-cell mediated anti-tumor immunity [23]. Of note, lenalidomide triggers NK-cell activation and increases antibody-dependent cell cytotoxicity in patients with FL [11], suggesting that combination of lenalidomide with a PD-1/PD-L1 inhibitor could have synergistic effects on NK cell anti-tumor activity in these patients.

Atezolizumab is a humanized immunoglobulin G1 mAb that targets PD-L1, inhibiting interaction with its receptors, PD-1 and B7-1 (also known as CD80) [24, 25]. Antibody-mediated PD-1 blockade has already been successfully exploited as a therapeutic strategy in several solid tumors and is currently being evaluated in hematologic malignancies [26]. The combination of obinutuzumab and atezolizumab has previously been shown to be well tolerated, with no new or unexpected safety signals, and with evidence of clinical activity in R/R FL in a phase Ib study [27].

We hypothesized that combining obinutuzumab, atezolizumab, and lenalidomide (G-atezo-len) in a triplet regimen could have the potential to enhance the anti-lymphoma immune response of the individual drugs. To explore this, we conducted a phase Ib/II study (BO29562; NCT02631577) to assess the safety and efficacy of the novel triplet combination of G-atezo-len as induction and maintenance therapy in patients with R/R FL. Data from the final analysis of this study are reported.

## Methods

### Patients

Patients aged ≥18 years were eligible for inclusion if they had histologically documented CD20-positive R/R FL (grade 1–3a), an Eastern Cooperative Oncology Group performance status of 0–2, at least 1 bi-dimensionally measurable lesion (>1.5 cm in its largest dimension by computed tomography [CT] scan or magnetic resonance imaging), and had received at least one prior anti-CD20 mAb-containing immunochemotherapy. Patients with grade 3b FL or a history of transformation of indolent disease to diffuse large B-cell lymphoma were excluded. To rule out the possible transformation, a core-needle biopsy was strongly recommended, but not mandatory, for patients with a biopsy taken more than 12 months prior to Day 1, Cycle 1 of treatment, or for patients who received anti-lymphoma treatment between the time of the most recent available biopsy and Day 1, Cycle 1. An overview of the full inclusion/exclusion criteria is provided in the Supplementary section.

### Study design

This was a phase Ib/II, open-label, multicenter, non-randomized study. The study comprised an initial 3 + 3 dose-escalation phase to determine the recommended phase II dose (RP2D) for lenalidomide when combined with fixed doses of obinutuzumab and atezolizumab in the G-atezo-len triplet regimen for induction treatment. The dose-escalation phase was followed by an expansion phase.

During the dose-escalation phase, patients received induction with 6, 28-day cycles of obinutuzumab 1000 mg intravenously (IV; Days 1, 8, 15 of Cycle 1; Day 1, Cycles 2–6), atezolizumab 840 mg IV (Days 1, 15, Cycles 2–6), and lenalidomide 15 or 20 mg orally (Days 1–21, Cycles 1–6). Patients enrolled in the subsequent expansion phase received the same G-atezo-len induction regimen as used in the dose-escalation phase, but with administration of lenalidomide at the established RP2D (20 mg) (Supplementary Fig. 1).

Patients who achieved a complete response (CR), partial response (PR), or stable disease (SD) at the end-of-induction (EOI) during the dose-escalation and expansion phases were eligible to receive extended dosing with G-atezo-len as maintenance treatment for up to 24 months or until disease progression or unacceptable toxicity. Maintenance treatment comprised obinutuzumab 1000 mg IV Day 1 every 2 months and atezolizumab 840 mg IV Day 1 and 2 every month with lenalidomide 10 mg orally (Days 1–21, months 1–12) started 8 weeks (±1 week) after Day 1 of Cycle 6.

The study was reviewed and approved by the ethics review boards of the relevant institutions and was conducted in accordance with the Declaration of Helsinki and Good Clinical Practice guidelines. All patients provided written informed consent.

### Study endpoints

The primary endpoint of phase Ib was to determine the RP2D for lenalidomide in combination with obinutuzumab and atezolizumab based on the incidence of dose-limiting toxicities (DLT; criteria provided in the Supplementary section) during Cycle 2 of study treatment.

In phase II, the primary endpoint was efficacy, defined as CR by positron emission tomography-computed tomography (PET-CT) and assessed by independent review committee (IRC; modified Lugano 2014 criteria) at EOI in the RP2D expansion cohort. Modifications to the standard Lugano criteria were as follows: for the designation of a PR on PET, criteria for CR or PR on CT scan had to be met; if bone marrow involvement was present at baseline, CR had to be confirmed with a negative bone marrow result at EOI.

Safety endpoints included evaluating the safety and tolerability of the G-atezo-len triplet regimen through the incidence of adverse events (AEs).

Secondary efficacy endpoints included: CR rate at EOI assessed by the investigator (INV; PET-CT) and by the IRC and INV (CT scans alone; standard Lugano 2014 criteria; [28] and objective response rate (ORR; defined as a CR or PR) at EOI assessed by the IRC and INV (PET-CT/CT scans alone).

Exploratory endpoints included: duration of response (DOR, all patients), progression-free survival (PFS), overall survival (OS), and ORR and CR rate at EOI among patients with and without progression of disease within 24 months (POD24) of first-line therapy.

### Assessments

All patients were closely monitored for AEs (criteria provided in the Supplementary section), with nature, frequency, severity, and timing of AEs reported throughout the study and for at least 35 days after the last dose of study treatment. Changes in vital signs, electrocardiograms, and clinical laboratory results during and following study treatment administration were recorded.

Response was determined by examination of PET and CT scans by the IRC and the INV using modified Lugano Response Criteria for Malignant Lymphoma. CT scans were performed at screening, at the EOI Cycle 2 (within 7 days prior to Day 1, Cycle 3), at 12, 18, and 24 months after initiation of induction treatment, and every 3 months post-treatment. Patients with radiographic signs of progression at the EOI Cycle 2 could continue to receive study treatment if the findings were considered to be due to pseudoprogression/tumor flare, but they were required to have a CT scan repeated 4–8 weeks later. PET-CT scans were performed at screening (within 35 days prior to Day 1, Cycle 1), at EOI in patients who had received ≥2 doses of induction treatment, and at 12 months after initiation of induction treatment if the PET-CT scan was positive at EOI. Bone marrow examinations were required at screening (within approximately 3 months prior to Day 1, Cycle 1) for staging purposes in all patients. If bone marrow infiltration was present at screening, a bone marrow biopsy was required at the EOI response assessment for all patients who may have achieved a CR, as defined per imaging methods. In patients with less than a CR at EOI, a bone marrow examination was also required to confirm a CR that was achieved after the EOI response assessment.

Minimal residual disease (MRD) was evaluated at EOI (at 10^−5^ sensitivity) using the Adaptive ClonoSEQ^®^ with next-generation sequencing platform (v2), with assessment of immunoglobulin heavy (IGH) and light chain (IGK), and BCL2-IGH alterations in DNA from peripheral blood mononuclear cells.

### Statistical analyses

The estimated sample size for the study was determined by the dose-escalation rules for a 3 + 3 algorithm. It was anticipated that enrolment of two dosing groups of 3–6 patients each, for a total of 6-12 patients with R/R FL, was required to establish the RP2D of lenalidomide during the dose-escalation phase. Approximately 40 patients were planned to be enrolled during the expansion phase. It was assumed that the PET-CT-defined CR rate with obinutuzumab-lenalidomide was ~40% in the R/R setting, as assessed by Cheson 2007 criteria [29]. A sample size of 40 patients was deemed sufficient to provide adequate precision for the point estimate and for the lower bound of the two-sided 90% confidence interval (CI) to rule out a clinically uninteresting probability of response of <46%, assuming an observed PET-CT-defined CR rate with G-atezo-len of 60%.

EOI response and safety analyses were performed on the primary population (cutoff date 23 October 2018), which included patients who received at least one dose of each study drug in the triplet combination. PFS was evaluated in the efficacy-evaluable population (patients in the lenalidomide 20 mg cohort who received all three drug components), at the time all patients had completed the 36-month visit or discontinued treatment (cutoff date 7 October 2020). Patients who received lenalidomide at the RP2D during the dose-escalation phase were pooled with patients in the expansion phase for the efficacy and safety analyses.

For all efficacy endpoints, point estimates are presented, along with the corresponding two-sided 90% Clopper-Pearson exact CIs. Patients without an EOI tumor assessment were considered to be non-responders. PFS and DOR were summarized descriptively using the Kaplan–Meier method. For the PFS analysis, data for patients without an event of interest were censored at the date of the last tumor assessment.

An interim analysis was conducted during the expansion phase of the study and data from the first 20 patients treated at the RP2D of lenalidomide were analyzed for PET-CT-defined CR at EOI. Enrolment was stopped early based on sponsor decision.

### Data sharing statement

Qualified researchers may request access to individual patient level data through the clinical study data request platform (https://vivli.org/). Further details on Roche’s criteria for eligible studies are available here (https://vivli.org/members/ourmembers/). For further details on Roche’s Global Policy on the Sharing of Clinical Information and how to request access to related clinical study documents, see here (https://www.roche.com/research_and_development/who_we_are_how_we_work/clinical_trials/our_commitment_to_data_sharing.htm).

## Results

### Patients

**Fig. 1:**
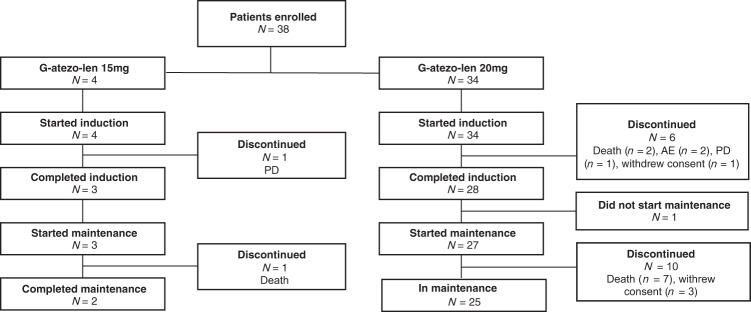
Patient disposition (final analysis). AE, adverse event; atezo, atezolizumab; G, obinutuzumab; L, lenalidomide; PD, progressive disease.

Following sponsor assessment, and unrelated to safety findings, enrolment was stopped after 38 patients; this was deemed a sufficient sample size to perform the planned benefit-risk assessment. At the time of the final analysis (cutoff date 7 October 2020), 38 patients (lenalidomide 15 mg, *n* = 4; 20 mg, *n* = 34) had completed the trial; 7 patients discontinued treatment during induction (progressive disease, *n* = 4 [death due to progressive disease in Cycle 1, *n* = 2]; AEs, *n* = 2; withdrawal of consent, *n* = 1) and 31 patients completed induction therapy. At the final cutoff date, 27 patients had completed maintenance treatment (Fig. 1).

Patient baseline characteristics for the safety population (*N* = 38) are summarized in Table 1. Median age was 61.5 (range 38–79) years; 79% of patients had Ann Arbor stage III/IV disease at diagnosis, and 26% had a high-risk Follicular Lymphoma International Prognostic Index (≥3); 47% had received ≥2 prior lines of therapy. Forty-five percent of patients were refractory to (did not respond to or progressed within 6 months of) their last line of treatment (29% to last line of anti-CD20 mAb) and 37% of patients had POD24 on first-line treatment.

**Table 1:** Baseline characteristics (safety population).

Characteristic, [*n* (%), unless stated]	Safety population (*N* = 38)
Median age, years (range)	61.5 (38–79)
Male	19 (50)
ECOG PS 0–1	38 (100)
Ann Arbor stage III/IV at diagnosis	30 (79)
FLIPI risk group [low (0–1); intermediate (2); high (≥3)]	6 (16); 22 (58); 10 (26)
Elevated LDH >1 × ULN	9 (24)
Prior lines of therapy [1; ≥2]	20 (53); 18 (47)
Prior treatment	
Bendamustine	12 (32)
CHOP	24 (63)
Obinutuzumab	1 (3)
Rituximab	35 (92)
Refractory to last line of treatment	17 (45)
Refractory to last line of anti-CD20 antibody	11 (29)
POD24 on first-line treatment	14 (37)
Bulky disease (≥7 cm)	6 (16)
Bone marrow infiltration	13 (35)*
Extranodal involvement	20 (53)

^*^N = 37.

*CHOP* cyclophosphamide, doxorubicin, vincristine, prednisone, *ECOG PS* Eastern Cooperative Oncology Group performance status; *FLIPI* Follicular Lymphoma International Prognostic Index, *LDH* lactate dehydrogenase, *POD24* progression of disease within 24 months, *ULN* upper limit of normal.

### Treatment exposure and follow-up

No DLTs were reported with either lenalidomide 15 mg or 20 mg during Cycle 2 of the dose-escalation phase; therefore, lenalidomide 20 mg was selected as the RP2D for expansion. At the time of the primary analysis, median follow-up was 30.0 months (range 2.7–32.1) in the lenalidomide 15 mg cohort and 14.2 months (range 0.6–24.8) in the lenalidomide 20 mg cohort.

At the time of final analysis, in the lenalidomide 20 mg cohort, the overall median treatment duration was 26.4 (range 0.4–29.5) months. The proportions of patients receiving >90% dose intensity during induction (*n* = 34) and maintenance (*n* = 28), respectively, were: obinutuzumab, 91.2% and 100%; atezolizumab, 71.9% and 85.7%; and lenalidomide, 76.5% and 85.7%; the respective proportions of patients receiving ≥75% dose intensity during induction and maintenance were obinutuzumab, 100% and 100%; atezolizumab, 90.6% and 85.7%; and lenalidomide, 88.2% and 92.9% (Supplementary Table 1).

### Efficacy

A total of 32 patients were evaluated for efficacy in the lenalidomide 20 mg cohort (Table 2; primary analysis). The IRC-assessed CR rate based on modified Lugano 2014 PET-CT criteria at EOI for the lenalidomide 20 mg cohort (primary efficacy endpoint) was 71.9%. The corresponding INV-assessed CR rate was 75.0%. Among patients who were double refractory (refractory to rituximab and alkylator), the CR rate (IRC-assessed; modified Lugano 2014 PET-CT criteria) was 66.7% (95% CI, 39.1–87.7; ORR 66.7%) compared with 75.0% (95% CI, 54.4–89.6; ORR 85.0%) among non-refractory patients (*P* = 0.6960; Supplementary Fig. 2A). Among POD24 patients, the CR rate (IRC-assessed; modified Lugano 2014 PET-CT criteria) was 50.0% (95% CI, 24.5–75.5; ORR 58.3%) compared with 85.0% (95% CI, 65.6–95.8; ORR 90.0%) among non-POD24 patients (*P* = 0.0493; Supplementary Fig. 2B).

**Table 2:** IRC- and INV-assessed response rates at EOI (lenalidomide 20 mg cohort, *n* = 32 evaluable patients; primary analysis).

	PET-CT scan (modified Lugano 2014)		CT-MRI scan (Lugano 2014)	
IRC-assessed	Patients, *n* (%)	90% CI	Patients, *n* (%)	90% CI
ORR	25 (78.1)	62.8–89.3	26 (81.3)	66.3–91.5
CR	23 (71.9)	56.1–84.5	10 (31.3)	18.0–47.2
PR	2 (6.3)		16 (50.0)	
SD	2 (6.3)		1 (3.1)	
PD	3 (9.4)		4 (12.5)	

INV-assessed				
ORR	27 (84.4)	69.9–93.6	28 (87.5)	73.6–95.6
CR	24 (75.0)	59.4–86.9	16 (50.0)	34.4–65.6
PR	3 (9.4)		12 (37.5)	
SD	1 (3.1)		1 (3.1)	
PD	1 (3.1)		1 (3.1)	

*CR* complete response, *CT* computed tomography, *EOI* end of induction, *INV* investigator, *IRC* independent review committee, *MRI* magnetic resonance imaging, *ORR* objective response rate, *PD* progressive disease, *PET* positron emission tomography, *PR* partial response, *SD* stable disease.

#### Response according to MRD status

A total of 22/28 MRD-evaluable patients had a circulating clone detected at baseline; 6 MRD-evaluable patients had no circulating clone at baseline. Of the 22 patients with a circulating clone at baseline, 21 were MRD evaluable at EOI (1 patient sample was not evaluable due to inadequate preparation of the sample).

Among the 21 MRD-evaluable patients at EOI, 16 (76.2%) were MRD negative; of these patients, 15 achieved a CR (93.8%) and 1 achieved a PR (6.3%), as determined by the IRC. Among the MRD-positive patients at EOI (*n* = 5), 1 patient (20.0%) had SD, 3 patients (60.0%) had disease progression, and 1 patient was not evaluable for response as determined by the IRC.

#### 36-month efficacy data

**Fig. 2:**
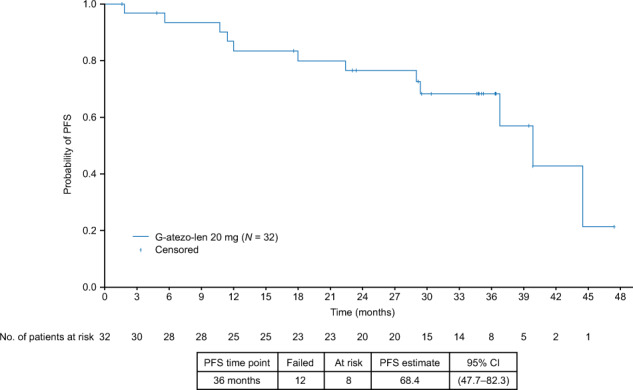
Kaplan–Meier estimate of INV-assessed progression-free survival amongst patients with relapsed/refractory follicular lymphoma (efficacy-evaluable population; 36-month cutoff: 7 October 2020). The median observation time was 35.9 months [range 3–47 months].

**Fig. 3:**
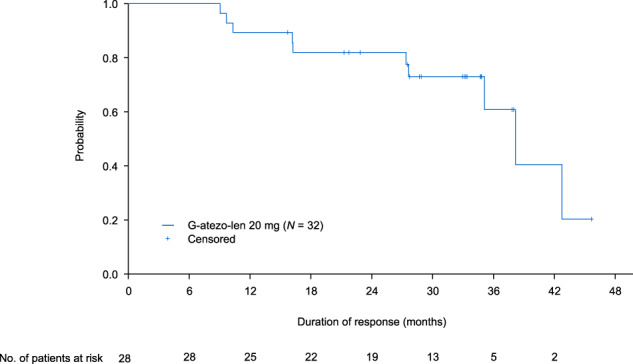
Duration of response* in 32 patients receiving G-atezo-len (INV-assessed, efficacy-evaluable population; 36-month cutoff: 7 October 2020). *Duration of response was defined as time from the first occurrence of a documented objective response to the time of disease progression or relapse, as determined by the investigator on the basis of CT scans alone or death from any cause, whichever occurred first.

In the efficacy-evaluable population, the 36-month PFS rate (data cutoff 7 October 2020; median observation time 35.9 months [range 3–47 months]) was 68.4% (95% CI, 48–82) (Fig. 2). There were 14 INV-assessed progression events. A total of 24 patients (75.0%) who received G-atezo-len as maintenance treatment had durable clinical responses (>1 year) and 18 patients had clinical response lasting >36 months (Fig. 3). The median DOR was 38 months (95% CI, 35-not estimable). The 36-month OS rate was 90.0% (95% CI, 72–97) (Supplementary Fig. 3). Two patient deaths in treatment Cycle 1 were considered by the investigators to be related to disease progression, although there was no radiologic or biopsy confirmation. These deaths were unrelated to atezolizumab treatment, which did not commence until Cycle 2.

### Safety

All treated patients (lenalidomide 15 mg and 20 mg cohorts) experienced ≥1 AE and 32 patients (84.2%) had a grade 3–5 AE (Table 3; final analysis). The incidence of AEs according to treatment period is summarized in Supplementary Table 2. All treated patients experienced at least 1 AE during induction, 31/38 patients (81.6%) who started maintenance experienced AEs during maintenance and 11/38 patients (28.9%) who started follow-up experienced AEs during the follow-up period.

**Table 3:** Summary of adverse events (final analysis).

Patient, *n* (%)	G-atezo-len 15 mg (*n* = 4)	G-atezo-len 20 mg (*n* = 34)	All patients (*N* = 38)
Any AE	4 (100.0)	34 (100.0)	38 (100.0)
Grade 3–5 AE	4 (100.0)	28 (82.4)	32 (84.2)
Grade 5 (fatal) AE^a^	0	2 (5.9)	2 (5.3)
Serious AE	2 (50.0)	16 (47.1)	18 (47.4)
AE leading to discontinuation of any study drug^b^	1 (25.0)	10 (29.4)	11 (28.9)
AE leading to study discontinuation^c^	0	2 (5.9)	2 (5.3)
AE leading to dose interruption of any treatment	4 (100.0)	30 (88.2)	34 (89.5)
Atezolizumab-related AESI (≥5%)^d^			
Hyperthyroidism	0	5 (14.7)	5 (13.2)
Hypothyroidism	0	4 (11.8)	4 (10.5)
ALT increased	1 (25.0)	2 (5.9)	3 (7.9)
AST increased	1 (25.0)	2 (5.9)	3 (7.9)
Lipase increased	0	3 (8.8)	3 (7.9)
Hepatocellular injury	0	2 (5.9)	2 (5.3)
Rash	0	2 (5.9)	2 (5.3)
Rash maculopapular	0	2 (5.9)	2 (5.3)
Squamous cell carcinoma	0	2 (5.9)	2 (5.3)
Pneumonitis	1 (25.0)	0	1 (2.6)
Bronchiolitis	1 (25.0)	0	1 (2.6)

*AE* adverse event, *AESI* adverse event of special interest, *ALT* alanine aminotransferase, *AST* aspartate aminotransferase, *atezo* atezolizumab, *G* obinutuzumab, *len* lenalidomide.

^a^The 2 fatal AEs were merkel carcinoma and sarcomatoid carcinoma; both unrelated to any study drug.

^b^Colitis, diarrhea, increased lipase, arthralgia, myalgia, acute myeloid leukemia, myelodysplastic syndrome, malignant lung neoplasm, ischemic stroke, lung disorder, pneumonitis, maculopapular rash, urticaria.

^c^The primary reason for discontinuation was death and the primary cause of death was a fatal AE (1 merkel carcinoma, 1 sarcomatoid carcinoma).

^d^≥5%, in either group (len 15 mg or 20 mg); all AESIs were grade ≤2 and resolved without any drug discontinuations.

The most common hematologic AEs (any grade) were neutropenia (17 patients [45%]), thrombocytopenia (10 patients [26%]) and anemia (7 patients [18%]) (Supplementary Table 3). Grade ≥3 hematologic AEs occurred in 27 patients (71%); the most common of these were neutropenia (16 patients [42%]), thrombocytopenia (7 [18%]) and anemia (3 [8%]). The most common non-hematologic AEs (any grade) were diarrhea (58%), constipation (40%), asthenia (37%), cough (37%) and infusion-related reactions (34%); these AEs were predominantly grade 1 or 2 (Supplementary Table 3). The most common non-hematologic grade ≥3 AEs were increased lipase (3 patients [8%]) and increased alanine aminotransferase (ALT, 2 patients [5%]).

A total of 28 serious AEs (SAEs) were reported in 18 (47.4%) patients (lung disorder was reported in two patients). Study drug withdrawal (permanent discontinuation of any treatment) due to an AE was reported in 11 (28.9%) patients who experienced a total of 13 AEs; these included colitis, diarrhea, increased lipase, arthralgia, myalgia, acute myeloid leukemia, malignant lung neoplasm, ischemic stroke, lung disorder, pneumonitis, maculopapular rash, and urticaria. The severity and timing of each of these permanent study drug discontinuations due to an AE (atezolizumab, 6 patients; lenalidomide, 3 patients; obinutuzumab, 3 patients) is summarized in Supplementary Table 4. Three of these AEs, acute myeloid leukemia, ischemic stroke, and increased lipase (2 incidences in the same patient), were categorized as grade 4 AEs; and four of these AEs (acute myeloid leukemia, ischemic stroke, lung disorder, and malignant lung neoplasm) were classed as SAEs. Three of these AEs resulted in permanent discontinuation of all three study drugs (acute myeloid leukemia, ischemic stroke, and malignant lung neoplasm), but not discontinuation from the study itself.

Overall, AEs led to dose modification/interruption of any study drug in 34 patients (89.5%). The most common events (≥10%) leading to dose modification/interruption were hyperthyroidism (18.4%) and hematologic toxicities including neutropenia (13.2%), and thrombocytopenia (10.5%). Two fatal AEs were reported; both were unrelated to any study drug (1 merkel carcinoma, 1 sarcomatoid carcinoma).

Reported adverse events of special interest (AESI) with obinutuzumab included 3 cases of second malignancies (grade 2 atypical fibroxanthoma, and grade 2 and grade 3 squamous cell carcinoma) which resolved following treatment. There were no cases of tumor lysis syndrome. The most common atezolizumab AESIs included hyperthyroidism (13%; based on laboratory abnormalities detected through frequent testing of thyroid hormones), hypothyroidism (11%), increased ALT and aspartate aminotransferase (both 8%), increased lipase (8%), hepatocellular injury, rash, maculopapular rash and squamous cell carcinoma (5% each). Two patients received hormone-replacement treatment for hypothyroidism.

## Discussion

In this phase Ib/II study, the chemotherapy-free triplet regimen G-atezo-len (lenalidomide 20 mg) demonstrated marked efficacy and an acceptable and manageable toxicity profile when used as induction and maintenance therapy in patients with R/R FL who had received at least one prior anti-CD20 mAb-containing immunochemotherapy regimen. The primary endpoint was met: G-atezo-len resulted in a CR rate at EOI of 71.9% and a 36-month PFS rate of 68.4%. Most responses were durable, with 18 patients experiencing clinical responses lasting longer than 36 months. The efficacy of the G-atezo-len triplet regimen was also reflected in high molecular response rates. Of 21 MRD-evaluable patients at EOI, 76% (16/21) were MRD negative, which was strongly associated with achievement of a CR (15/16 MRD-negative patients; 93.8%).

A promising CR rate of 67% was reported for the sub-group of patients with double-refractory disease. Among the 12 patients with POD24, the CR rate was 50% compared with 85% for patients without POD24 (*N* = 20). Of note, two patients died early in Cycle 1 of treatment, which is quite uncommon for true FL patients. These deaths were unrelated to atezolizumab because it was not administered until the start of Cycle 2. The authors consider that these deaths may be a consequence of misdiagnosed histological transformation in these patients at study entry, as there was no mandatory histological biopsy confirmation at baseline. It has been recently shown that the negative prognostic impact of POD24 is strongly related to histological transformation [30] and that anti-CD20 len-based combinations are not an adequate treatment option in this setting [31]. Given the small sample size, this may have affected response and PFS findings in our series.

Our results with the G-atezo-len regimen showing a 3-year PFS rate of 68.4% look encouraging. Prior series evaluating anti-CD20 mAb + len reported a 65% 2-year PFS rate for the G-len regimen [18] and 53% for the R2 regimen [12]. Additionally, pivotal phase II studies in R/R FL, have reported PFS rates for the PI3K inhibitors, idelalisib and duvelisib, of 47% (IRC-assessed PFS rate at 48 weeks) [5] and 62% (IRC-assessed PFS rate at 6 months) [4], respectively. However, patients in the PI3K inhibitor clinical trials were high-risk, having received a median of ≥3 lines of prior therapy and in some cases, were double refractory to rituximab and/or chemotherapy/radioimmunotherapy. Additionally, any cross-trial comparison is challenging due to differences between studies in dosing regimen, patient population/inclusion criteria, endpoints, and response criteria (PET based in our series).

The acceptable and manageable toxicity profile of the G-atezo-len regimen in the current study was substantiated by the high proportion of patients (>85%) receiving >75% dose intensity during induction and maintenance. Furthermore, the safety and tolerability profile of G-atezo-len was generally consistent with the known profiles for the individual drugs and the double combination regimens, obinutuzumab plus lenalidomide, and obinutuzumab plus atezolizumab, with no new safety signals identified [18, 27, 32–36]. Most AEs in the study were manageable with appropriate medical care or dose modifications. The majority of AEs were grade 1–2, and most grade 3–5 AEs and all SAEs were isolated events. The rate of permanent discontinuation of any study drug due to AEs was 29%. The incidence and severity of known AEs associated with obinutuzumab (i.e., infusion-related reactions, hypersensitivity reactions, neutropenia, thrombocytopenia) was consistent with the known safety profile of obinutuzumab [6, 34]. AESI to obinutuzumab were uncommon; there were 2 reports of grade 2-second malignancy, which subsequently resolved following treatment, and there were no events of tumor lysis syndrome. Among the AESIs associated with atezolizumab were hyperthyroidism, hypothyroidism, elevated hepatic transaminases and lipase, hepatocellular injury, and maculopapular rash. The incidence of thyroid-related AEs, particularly hyperthyroidism, which was often transient but, in some cases, clinically relevant in this study, was slightly higher than expected. However, it should be noted that the study protocol mandated a high frequency of laboratory testing that may have contributed to this increased incidence and that thyroid dysfunction is a well-documented side-effect of checkpoint inhibitors, including atezolizumab [37].

Of note, despite some safety concerns of lenalidomide in combination with PD-1/PD-L1 inhibitors in two phase III trials in patients with multiple myeloma, which reported excessive and unpredictable toxicity necessitating trial discontinuation, and an increased death rate, following treatment with an anti-PD-1 mAb and immunomodulatory agent (KEYNOTE-185: pembrolizumab/lenalidomide/dexamethasone; KEYNOTE-183: pembrolizumab/pomalidomide/dexamethasone) [36, 38], the addition of atezolizumab to G-len did not lead to an unacceptable increase in the incidence of immune-based toxicities in R/R FL patients in our study.

## Conclusions

In conclusion, this phase Ib/II study provides evidence of activity for the immunomodulatory triplet combination of G-atezo-len in R/R FL. The high rate of MRD negativity observed in our study is encouraging given the previously reported prognostic value of MRD status at EOI for long-term PFS in patients with R/R FL (GADOLIN trial) [39]. Although the sample size is too limited to draw definitive conclusions, data from this final analysis suggest that the addition of atezo to G-len may contribute to the durability of response.

## Supplementary information

Supplementary Material
