# Supplementary material for: Obinutuzumab-atezolizumab-lenalidomide for the treatment of patients with relapsed/refractory follicular lymphoma: final analysis of a Phase Ib/II trial
Source: Blood Cancer J. 2021 Aug 20;11(8):147. doi: 10.1038/s41408-021-00539-8 (PMC8379261; doi:10.1038/s41408-021-00539-8)

**Supplementary information**

**Safety parameters**

**Dose limiting toxicity (DLT)**

In this study a DLT was defined as:

1. An adverse event (AE) of any grade that leads to a delay of ≥14 days at the start of the next treatment cycle.
2. Grade 3 or 4 neutropenia in the presence of sustained fever of >38°C (lasting >5 days) or a documented infection.
3. Grade 4 neutropenia or thrombocytopenia lasting >7 days.
4. Grade 3 or 4 thrombocytopenia that results in significant bleeding per investigator judgment.
5. Grade 3 or 4 infusion-related reactions (IRRs).
6. Grade 3 diarrhea that responds to therapy within 72 hours.
7. Grade 3 nausea or vomiting that occurs in the absence of premedication and responds to adequate therapy within 72 hours.
8. Other toxicities considered to be clinically relevant and related to study treatment (as determined by the investigator and the medical monitor).

**Adverse events (AEs)**

An AE can be:

1. Any unfavorable and unintended sign (including an abnormal laboratory finding), symptom or disease temporally associated with the use of a medicinal product (whether considered related to the medicinal product or not).
2. Any new disease or exacerbation of an existing disease (a worsening in the character, frequency, or severity of a known condition).
3. Recurrence of an intermittent medical condition (e.g., headache) not present at baseline.
4. Any deterioration in a laboratory value or other clinical test (e.g., electrocardiogram, X-ray) associated with symptoms or leading to changes in study treatment, concomitant treatment or discontinuation from study treatment.
5. Any AEs related to a protocol-mandated intervention, including those that occur prior to assignment of study treatment (e.g., screening invasive procedures such as biopsies).

**Serious AEs (SAEs)**

SAEs were required to be reported by the investigator to the sponsor immediately (no more than 24 hours after learning of the event). Criteria for a SAE includes any AE that:

1. Is fatal (causes or leads to death).
2. Is life-threatening (is determined by the investigator as placing the patient at immediate risk of death).
3. Requires or prolongs in-patient hospitalization.
4. Results in persistent or significant disability/incapacity (significant disruption of the patient’s ability to conduct normal life functions).
5. Results in a congenital anomaly/birth defect in a neonate/infant born to a mother exposed to study treatment.
6. Is judged by the investigator to be a significant medical event, which may jeopardize the patient or requires medical/surgical intervention to prevent one of the outcomes mentioned previously.

Additionally, second primary malignancies, regardless of causal relationship to study treatment occurring at any time during the study (from the time of signing the informed consent form up to 3 years after the last dose of lenalidomide) were also classified as SAEs.

**AEs of special interest (AESIs)**

AESIs including cases of potential drug-induced liver injury that include elevated alanine aminotransferase or aspartate transaminase in combination with elevated bilirubin or clinical jaundice (as defined by Hy’s law) and suspected transmission of an infectious agent by the study treatment, were required to be reported by the investigator to the sponsor immediately (no more than 24 hours after learning of the event).

AESIs to obinutuzumab are tumor lysis syndrome of any grade (irrespective of causality) and second malignancies. AESIs to atezolizumab are pneumonitis, colitis, adrenal insufficiency, diabetes mellitus, pancreatitis, hypothyroidism, hyperthyroidism, hepatitis, Guillain-Barré syndrome, myasthenia gravis, nephritis, meningitis, encephalitis, myositis, rhabdomyolysis, rash, ocular inflammatory toxicity, vasculitis, hypophysitis, myocarditis, severe cutaneous reaction and hemolytic anemia (all immune-related), IRRs, systemic immune activation and autoimmune hemolytic anemia.

**Selected AEs**

Defined as AEs for which additional data collection or analyses were performed; including thrombocytopenia, acute thrombocytopenia (events occurring within 24 hours of obinutuzumab infusion), hepatitis B reactivation, cardiac events, IRRs, all infections (including progressive multifocal leukoencephalopathy), neutropenia, prolonged neutropenia (neutropenia <1000 cells/µL that does not resolve after 28 days without obinutuzumab treatment), late-onset neutropenia (neutropenia <1000 cells/µL occurring ≥28 days after obinutuzumab treatment has been completed or stopped) and gastro-intestinal perforation.

**Key inclusion criteria**

1. Age ≥18 years.
2. Relapsed/refractory (R/R) follicular lymphoma (FL) after treatment with ≥1 prior immunochemotherapy regimen that included an anti-CD20 monoclonal antibody and for which no other more appropriate treatment option exists (as determined by the investigator).
3. Histologically documented, CD20-positive and fluorodeoxyglucose-avid (ie, positron emission tomography-positive lymphoma) with ≥1 bi-dimensionally measurable lesion.
4. Eastern Cooperative Oncology Group performance status of 0-2.
5. Availability of a representative tumor specimen and the corresponding pathology report for retrospective central confirmation of the diagnosis of FL.
6. Agreement to comply with all local requirements of the lenalidomide risk minimization plan.
7. *For women of childbearing potential:* agreement to remain abstinent or to use contraceptive methods that result in a failure rate of <1% per year for at least 28 days prior to Day 1 of Cycle 1 (D1C1), during the treatment period and for at least 18 months after the last dose of study treatment.
8. *For men*: agreement to remain abstinent or to use contraceptive measures during the treatment period and for at least 3 months after the last treatment, and agreement to refrain from donating sperm during this same period.

**Exclusion criteria**

1. Grade 3b FL.
2. History of transformation of indolent disease to diffuse large B-cell lymphoma.
3. Known CD20-negative status at relapse or progression.
4. Central nervous system lymphoma or leptomeningeal infiltration.
5. Prior allogeneic stem cell transplantation (ASCT) or completion of ASCT within 100 days prior to D1C1.
6. History of resistance to lenalidomide or response duration of <1 year (for patients who had response to a prior lenalidomide-containing regimen).
7. Prior anticancer therapy including: lenalidomide, fludarabine or alemtuzumab within 12 months prior to D1C1; radioimmunoconjugate within 12 weeks prior to D1C1; monoclonal antibody or antibody drug conjugate within 4 weeks prior to D1C1; radiotherapy, chemotherapy, hormonal therapy, or targeted small molecule therapy within 2 weeks prior to D1C1; or anti-programmed death-1 (anti-PD-1), anti-programmed death-ligand 1 (anti-PD-L1), anti-cytotoxic T-lymphocyte-associated protein 4 (CTLA4), anti-CD137/41-BB agonist, or anti-CD40 agonist antibodies.
8. Treatment with systemic immunosuppressive medications, including, but not limited to, prednisone, azathioprine, methotrexate, thalidomide, or anti-tumor necrosis factor agents within 2 weeks prior to D1C1.
9. History of solid organ transplantation.
10. History of severe allergic or anaphylactic reaction to humanized, chimeric, or murine monoclonal antibodies.
11. History of erythema multiforme, grade ≥3 rash, or blistering following prior treatment with immunomodulatory derivatives such as thalidomide and lenalidomide.
12. Active infection or a major episode of infection requiring treatment with IV antibiotics within 4 weeks of D1C1.
13. Testing positive for hepatitis B surface agent, total hepatitis B core antibody, or hepatitis C virus antibody at screening.
14. Known history of human immunodeficiency virus (HIV)-positive status.
15. Progressive multifocal leukoencephalopathy or autoimmune disease.
16. Vaccination with a live virus vaccine or live attenuated vaccine within 28 days prior to D1C1.
17. Pre-existing grade >1 neuropathy.
18. Major surgical procedure other than for diagnosis within 28 days prior to D1C1.
19. Inadequate hematologic function, renal function, or liver function.
20. Pregnant or lactating women.
21. Life expectancy <3 months.

**Supplementary Table 1.** Treatment exposure during induction and maintenance (lenalidomide 20 mg cohort; final analysis)

|  | **G** | **Atezo** | **Len** |
| --- | --- | --- | --- |
| **Induction** | *N* = 34 | *N* = 34 | *N* = 34 |
| Median number of cycles received (range) | 6 (1-6) | 5 (0-5) | 6 (1-6) |
| Median dose intensity, % (range) | 100 (88-100) | 100 (30-100)* | 99.2 (14-102) |
| Patients who received <75% dose, *n* (%) | 0 | 3 (9.4) | 4 (11.8) |
| Patients who received 75-90% dose, *n* (%) | 3 (8.8) | 6 (18.8) | 4 (11.8) |
| Patients who received >90% dose, *n* (%) | 31 (91.2) | 23 (71.9) | 26 (76.5) |
| **Maintenance** | *N* = 28 | *N* = 28 | *N* = 28 |
| Median number of cycles received (range) | 12 (2-12) | 22.5 (0-24) | 12 (0-12) |
| Median dose intensity, % (range) | 100 (92-100) | 100 (0-100) | 99.1 (0-100) |
| Patients who received <75% dose, *n* (%) | 0 | 4 (14.3) | 2 (7.1) |
| Patients who received 75-90% dose, *n* (%) | 0 | 0 | 2 (7.1) |
| Patients who received >90% dose, *n* (%) | 28 (100) | 24 (85.7) | 24 (85.7) |

**N* = 32.

Atezo, atezolizumab; G, obinutuzumab; Len, lenalidomide.

**Supplementary Table 2.** Summary of adverse events by treatment period (data cutoff 07 October 2020)

|  | **Induction phase** | | | **Post-induction phase** | | | **Follow-up** | | |
| --- | --- | --- | --- | --- | --- | --- | --- | --- | --- |
| **Patient, n (%)** | **G-atezo-len 15 mg (*n* = 4)** | **G-atezo-len 20 mg (*n* =34)** | **All patients (*N* = 38)** | **G-atezo-len 15 mg (*n* = 4)** | **G-atezo-len 20 mg (*n* = 34)** | **All patients (*N* = 38)** | **G-atezo-len 15 mg (*n* = 4)** | **G-atezo-len 20 mg *(n* = 34)** | **All patients (*N* = 38)** |
| Any AE | 4 (100.0) | 34 (100.0) | 38 (100.0) | 3 (75.0) | 28 (82.4) | 31 (81.6) | 1 (25.0) | 10 (29.4) | 11 (28.9) |
| Grade 3-5 AE | 3 (75.0) | 21 (61.8) | 24 (63.2) | 2 (50.0) | 16 (47.1) | 18 (47.4) | 1 (25.0) | 7 (20.6) | 8 (21.1) |
| Grade 5 (fatal) AE | 0 | 0 | 0 | 0 | 0 | 0 | 0 | 2 (5.9) | 2 (5.3) |
| Serious AE | 2 (50.0) | 6 (17.6) | 8 (21.1) | 0 | 9 (26.5) | 9 (23.7) | 0 | 5 (14.7) | 5 (13.2) |
| AE leading to discontinuation of any study drug | 0 | 5 (14.7) | 5 (13.2) | 1 (25.0) | 4 (11.8) | 5 (13.2) | 0 | 2 (5.9) | 2 (5.3) |
| AE leading to study discontinuation | 0 | 0 | 0 | 0 | 0 | 0 | 0 | 0 | 0 |
| Any AE related to G | 2 (50.0) | 29 (85.3) | 31 (81.6) | 1 (25.0) | 22 (64.7) | 23 (60.5) | 0 | 1 (2.9) | 1 (2.6) |
| Grade 3-5 AE | 0 | 14 (41.2) | 14 (36.8) | 0 | 7 (20.6) | 7 (18.4) | 0 | 1 (2.9) | 1 (2.6) |
| Serious AE | 0 | 2 (5.9) | 2 (5.3) | 0 | 2 (5.9) | 2 (5.3) | 0 | 0 | 0 |
| Any AE related to atezo | 2 (50.0) | 26 (76.5) | 28 (73.7) | 2 (50.0) | 20 (58.8) | 22 (57.9) | 0 | 2 (5.9) | 2 (5.3) |
| Grade 3-5 AE | 0 | 7 (20.6) | 7 (18.4) | 0 | 5 (14.7) | 5 (13.2) | 0 | 1 (2.9) | 1 (2.6) |
| Serious AE | 0 | 1 (2.9) | 1 (2.6) | 0 | 2 (5.9) | 2 (5.3) | 0 | 1 (2.9) | 1 (2.6) |
| Any AE related to len | 4 (100.0) | 30 (88.2) | 34 (89.5) | 2 (50.0) | 20 (58.8) | 22 (57.9) | 0 | 2 (5.9) | 2 (5.3) |
| Grade 3-5 AE | 1 (25.0) | 16 (47.1) | 17 (44.7) | 0 | 5 (14.7) | 5 (13.2) | 0 | 1 (2.9) | 1 (2.6) |
| Serious AE | 0 | 4 (11.8) | 4 (10.5) | 0 | 2 (5.9) | 2 (5.3) | 0 | 1 (2.9) | 1 (2.6) |

AE, adverse event; atezo, atezolizumab; G, obinutuzumab; len, lenalidomide

**Supplementary Table 3.** Most common hematologic and non-hematologic adverse events (any grade and grade ≥3)* (final analysis)

|  | **G-atezo-len 15 mg (*n* = 4)** | | **G-atezo-len 20 mg (*n* = 34)** | | **All patients (*N* = 38)** | |
| --- | --- | --- | --- | --- | --- | --- |
| **Patients, *n* (%)** | **Any grade** | **Grade ≥3** | **Any grade** | **Grade ≥3** | **Any grade** | **Grade ≥3** |
| **Hematologic AEs** | |  |  |  |  |  |
| Neutropenia | 3 (75.0) | 2 (50.0) | 14 (41.2) | 14 (41.2) | 17 (44.7) | 16 (42.1) |
| Thrombocytopenia | 1 (25.0) | 0 | 9 (26.5) | 7 (20.6) | 10 (26.3) | 7 (18.4) |
| Anemia | 1 (25.0) | 1 (25.0) | 6 (17.6) | 2 (5.9) | 7 (18.4) | 3 (7.9) |
| **Non-hematologic AEs** | |  |  |  |  |  |
| Diarrhea | 3 (75.0) | 0 | 19 (55.9) | 1 (2.9) | 22 (57.9) | 1 (2.6) |
| Asthenia | 0 | 0 | 14 (41.2) | 1 (2.9) | 14 (36.8) | 1 (2.6) |
| Cough | 2 (50.0) | 0 | 12 (35.3) | 0 | 14 (36.8) | 0 |
| Constipation | 3 (75.0) | 0 | 12 (35.3) | 0 | 15 (39.5) | 0 |
| Infusion-related reactions^†^ | 1 (25.0) | 0 | 12 (35.3) | 0 | 13 (34.2) | 0 |
| Abdominal pain | 2 (50.0) | 1 (25.0) | 8 (23.5) | 0 | 10 (26.3) | 1 (2.6) |
| Fatigue | 1 (25.0) | 0 | 8 (23.5) | 0 | 9 (23.7) | 0 |
| Hyperthyroidism | 1 (25.0) | 0 | 7 (20.6) | 0 | 8 (21.1) | 0 |
| Nausea | 4 (100.0) | 1 (25.0) | 4 (11.8) | 0 | 8 (21.1) | 1 (2.6) |
| Pyrexia | 3 (75.0) | 0 | 5 (14.7) | 0 | 8 (21.1) | 0 |
| Lipase increased | 1 (25.0) | 1 (25.0) | 4 (11.8) | 2 (5.9) | 5 (13.2) | 3 (7.9) |
| ALT increased | 1 (25.0) | 1 (25.0) | 2 (5.9) | 1 (2.9) | 3 (7.9) | 2 (5.3) |
|  |  |  |  |  |  |  |

*The table summarizes the most common AEs (any grade) occurring in ≥20% of patients overall along with their grade ≥3 incidence, and also summarizes the most common grade ≥3 AEs occurring in ≥5% patients overall.

^†^Infusion-related reactions were defined as any related AEs occurring during or within 24 hours of infusion of study medication.

AE, adverse event; ALT, alanine aminotransferase; atezo, atezolizumab; G, obinutuzumab; len, lenalidomide.

**Supplementary Table 4.** Summary of study drug withdrawal (permanent discontinuation of any treatment) due to an adverse event

| **AE** | **AE intensity and outcome*** | **Study drug discontinued** | **Timing of last drug administration** |
| --- | --- | --- | --- |
|  |  |  |  |
| Diarrhea | Grade 2, resolved | Atezolizumab^†^  Lenalidomide^†^ | Day 2, Maint Month 12  Day 20, Maint Month 11 |
| Arthralgia | Grade 2, unresolved | Atezolizumab^†^ | Day 2, Maint Month 6 |
| Myalgia | Grade 1 AE, resolved | Atezolizumab^†^ | Day 15, Cycle 5 |
| Myelodysplastic syndrome | Grade 4 SAE with hospitalization, unresolved | Obinutuzumab^†^  Atezolizumab^†^  Lenalidomide^†^ | Day 1, Maint Month 9  Day 2, Maint Month 10  Day 2, Maint Month 9 |
| Ischemic stroke | Grade 4 SAE with hospitalization, resolved with sequelae | Obinutuzumab^‡^  Atezolizumab^‡^  Lenalidomide^†^ | Day 1, Cycle 3  Day 1, Cycle 3  Day 14, Cycle 3 |
| Pneumonitis | Grade 2 AE, resolved | Atezolizumab^†^ | Day 2, Maint Month 5 |
| Maculopapular rash | Grade 2, unresolved | Lenalidomide^†^ | Day 8, Cycle 4 |
| Urticaria | Grade 2, resolved | Lenalidomide^†^ | Day 11, Cycle 2 |
| Lung disorder | Grade 3, resolved | Atezolizumab^†^ | Day 2, Maint Month 23 |
| Colitis | Grade 2, resolved | Atezolizumab^†^ | Day 1, Cycle 4 |
| Lung neoplasm malignant | Grade 3, unresolved | Obinutuzumab    Atezolizumab  Lenalidomide | Day 1, Maint Month 11  Day 2, Maint Month 11  Day 22, Maint Month 22 |
| Lipase increased | Grade 4, resolved | Atezolizumab  Obinutuzumab | Day 1, Cycle 6  Day 1, Maint Month 3 |

*At the clinical cutoff date (7 October 2020).

^†^AE considered to be related to the discontinued study drug.

^‡^Ischemic stroke considered unrelated to obinutuzumab and atezolizumab.

AE, adverse event; Maint, maintenance; SAE, serious adverse event.

**Supplementary Figure 1.** Study design


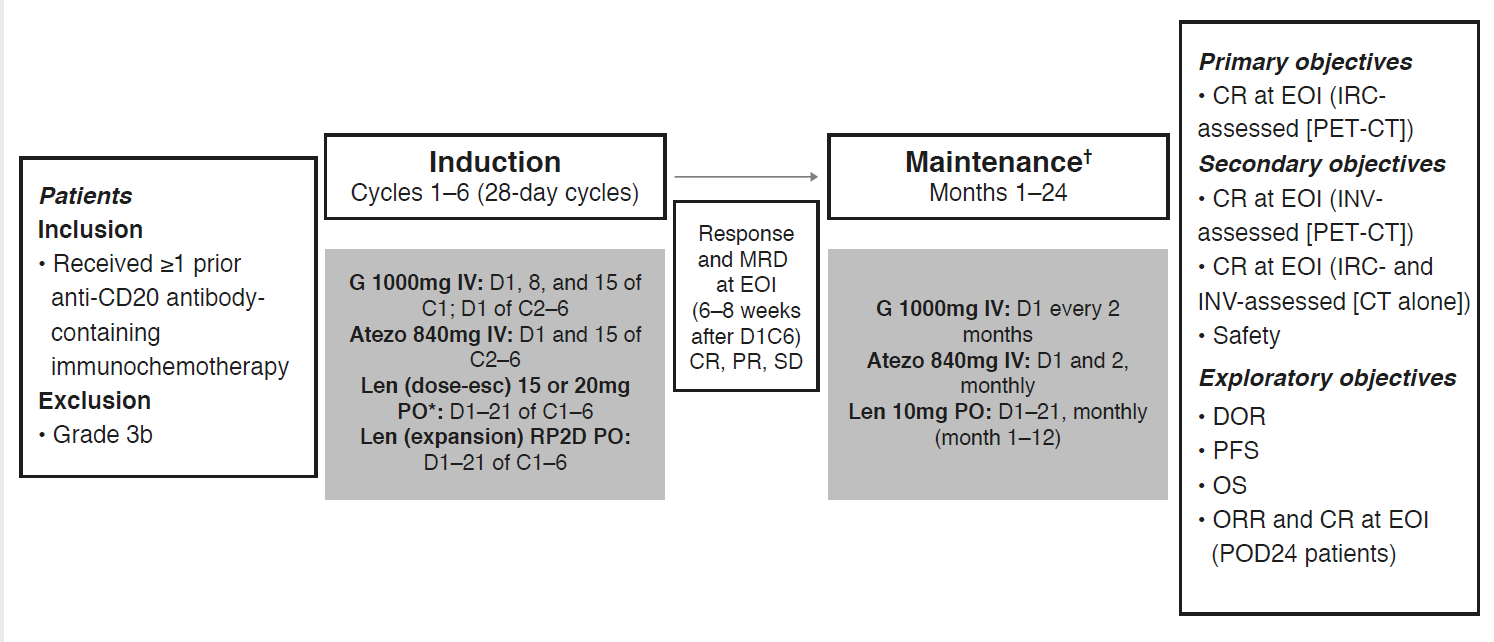


*Len dose could be de-escalated to 10mg during the escalation phase; ^†^Maintenance treatment started 8 weeks (± 1 week) after D1C6.

Atezo, atezolizumab; C, cycle; CR, complete response; CT, computed tomography; D, day; DOR, duration of response; EOI, end of induction; esc, escalation; G, obinutuzumab; INV, investigator; IRC, independent review committee; IV, intravenous; Len, lenalidomide; MRD, minimal residual disease; ORR, objective response rate; OS, overall survival; PET, positron emission tomography; PO, oral; PFS, progression-free survival; POD24, progression of disease within 24 months; PR, partial response; RP2D, recommended phase II dose; SD, stable disease.

**Supplementary Figure 2.** Independent review committee-assessed response rates in (A) double-refractory patients and (B) patients with progressive disease within 24 months from initial diagnosis


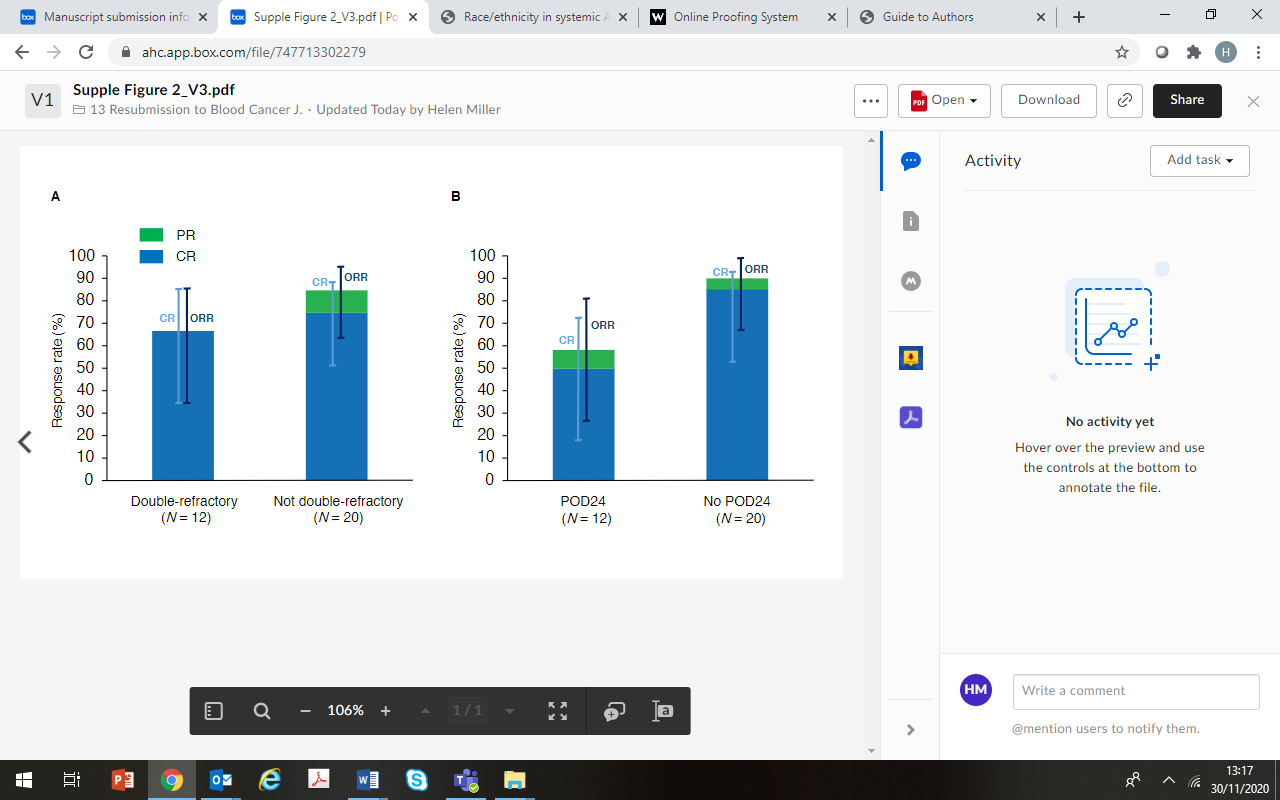


CR, complete response; ORR, overall response rate; POD24, progressive disease within 24 months; PR, partial response.

**Supplementary Fig 3.** Kaplan-Meier estimate of overall survival amongst patients with relapsed/refractory follicular lymphoma (ITT population; 36-month cutoff: 7 October 2020).


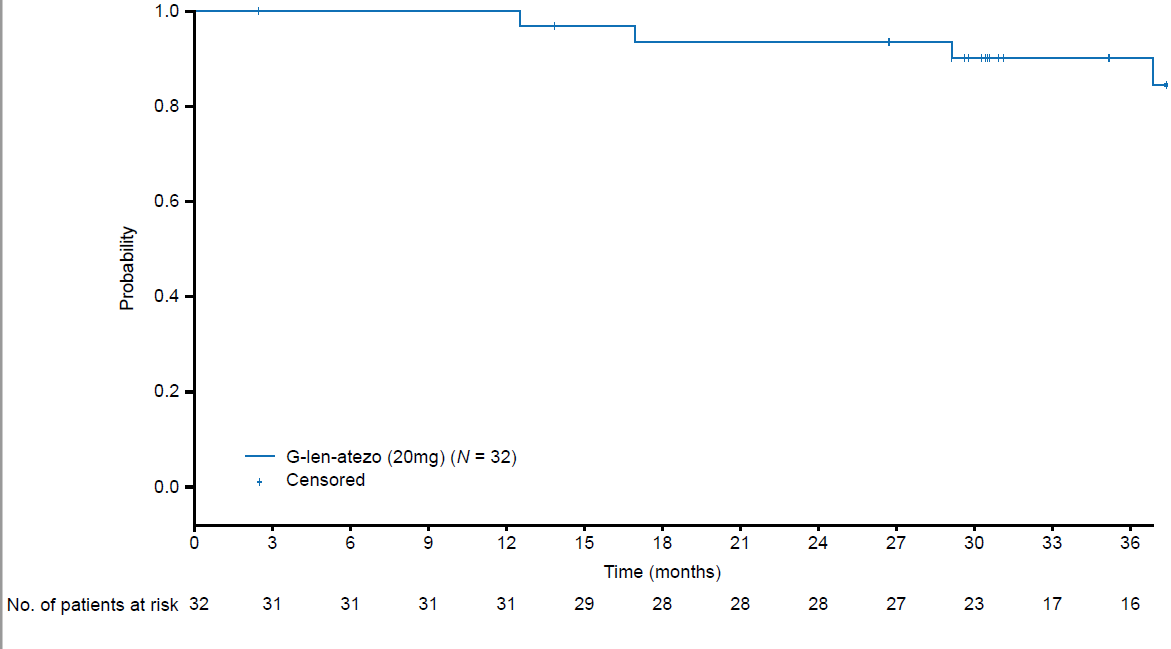

Supplement: Supplementary file 1 — Supplementary Material [file 41408_2021_539_MOESM1_ESM.docx]
